# Malaria risk mapping among children under five in Togo

**DOI:** 10.1038/s41598-024-58287-1

**Published:** 2024-04-08

**Authors:** Gountante Kombate, Issouf Kone, Bili Douti, Kamba André-Marie Soubeiga, Diederick E. Grobbee, Marianne A. B. van der Sande

**Affiliations:** 1 Ministry of Health and Public Hygiene, Lomé, Togo; 2 Interdisciplinary Research Laboratory in Social Health Sciences University Joseph Ki-Zerbo, Ouagadougou, Burkina Faso; 3 African School of Economics (ASE), Cotonou, Benin; 4 Ministry of Health and Public Hygiene, Lomé, Togo; 5 Interdisciplinary Research Laboratory in Social and University Joseph Ki-Zerbo, Ouagadougou, Burkina Faso; 6 Global Public Health, Julius Centre, University Medical Centre Utrecht, Utrecht, The Netherlands; 7 Department of Public Health, Institute of Tropical Medicine, Antwerp, Belgium

**Keywords:** Epidemiology, Infectious diseases

## Abstract

Malaria is a major health threat in sub-Sahara Africa, especially for children under five. However, there is considerable heterogeneity between areas in malaria risk reported, associated with environmental and climatic. We used data from Togo to explore spatial patterns of malaria incidence. Geospatial covariate datasets, including climatic and environmental variables from the 2017 Malaria Indicator Survey in Togo, were used for this study. The association between malaria incidence and ecological predictors was assessed using three regression techniques, namely the Ordinary Least Squares (OLS), spatial lag model (SLM), and spatial error model (SEM). A total of 171 clusters were included in the survey and provided data on environmental and climate variables. Spatial autocorrelation showed that the distribution of malaria incidence was not random and revealed significant spatial clustering. Mean temperature, precipitation, aridity and proximity to water bodies showed a significant and direct association with malaria incidence rate in the SLM model, which best fitted the data according to AIC. Five malaria incidence hotspots were identified. Malaria incidence is spatially clustered in Togo associated with climatic and environmental factors. The results can contribute to the development of specific malaria control plans taking geographical variation into consideration and targeting transmission hotspots.

## Introduction

Malaria in children under the age of five is a major cause of morbidity and mortality worldwide. Despite efforts to combat the disease, the number of cases is rising, and has been estimated at around 232 million in 2019, 245 million in 2020 and 247 million in 2021. Sub-Saharan African countries have the highest rates of infection, with children under the age of five most affected^1^.

For example, in Togo, 2,136,877 confirmed cases and 1275 deaths were recorded in 2020 on a population of about 8 million inhabitants. Children under five years of age represented 31.6% of cases^2,3^. According to the distribution of cases reported in the country by the National Malaria Control Program (PNLP), malaria infection is regionally heterogeneous: in 2019, malaria prevalence was 18% in the Lomé commune region, 39% in the Plateaux region and 42% in the Kara region^4^. A recent study in Togo on spatio-temporal analysis used district-level health system data (2008–2017) to analyze the trend and change in malaria incidence^5^. Other studies have explored the burden of malaria in children under five^6–9^ at the regional level^4^ using individual and household level factors, but contextual factors such as the climate and environmental may play an important role in malaria transmission also^10^. Thus, the spatial variation observed may be related to different ecological conditions, variations in health care systems, and socioeconomic differences or intervention coverage^11^. Effective interventions and prevention efforts could be improved by deepening our understanding of the spatial variation and related ecological factors underlying malaria transmission and risk of disease^12^.

Several environmental and ecological factors are known to influence malaria transmission; of which the main ones are precipitation and temperature^12,13^. Precipitation is directly related to rainfall and influences the biological dynamics of the Anopheles mosquito vectors of malaria parasites. Rainfall, especially when it is heavy, washes away much of the breeding sites of these mosquitoes, while temperature determines during how much time mosquito larvae can develop in the environment and parasites in the vector. Environmental change caused by the construction of dams and irrigation systems can influence the type and distribution of mosquitoes^13,14^. Environmental and ecological factors can lead to local transmission hotspots, defined as geographical areas within a larger transmission zone in which the intensity incidence is significantly higher than the average level in the surrounding area. In contrast, there are cold spots of malaria, defined as a location of cases where the intensity of transmission incidence is significantly lower than expected^15^.

Hotspots are the main reservoirs of persistent malaria transmission and are associated with higher vector density, sporozoite prevalence, and malaria incidence than in neighboring areas^16^. Malaria hotspots occur mostly in poor, tropical and subtropical areas of the world, with Africa being the most affected. The main risk factors of malaria hotspots are associated with local climatic and environmental conditions, such as proximity to vector breeding sites, precipitation^15^, vegetation cover, temperatures, housing conditions^17,18^, net use and household occupancy^19^. Nevertheless, the exact role of these factors in the formation of malaria hotspots is still under debate^20–22^. Several African countries have observed the persistence of malaria hotspots after an overall reduction in malaria transmission^21,22^. Lack of knowledge about malaria hotspots can therefore undermine the effectiveness of intervention programs. Identifying these hotspots is crucial to achieving the sustainable development goal of zero malaria incidence in a given area by 2030^23,24^ particularly in Togo.

The aim of this study was to use geospatial techniques to identify malaria hotspots on the basis of environmental and climatic factors in a Togolese context, as availability of national data from the 2017 Malaria Indicators Survey provided a unique opportunity to assess spatial and temporal patterns of malaria transmission.

## Methods

### Study areas

**Figure 1:**
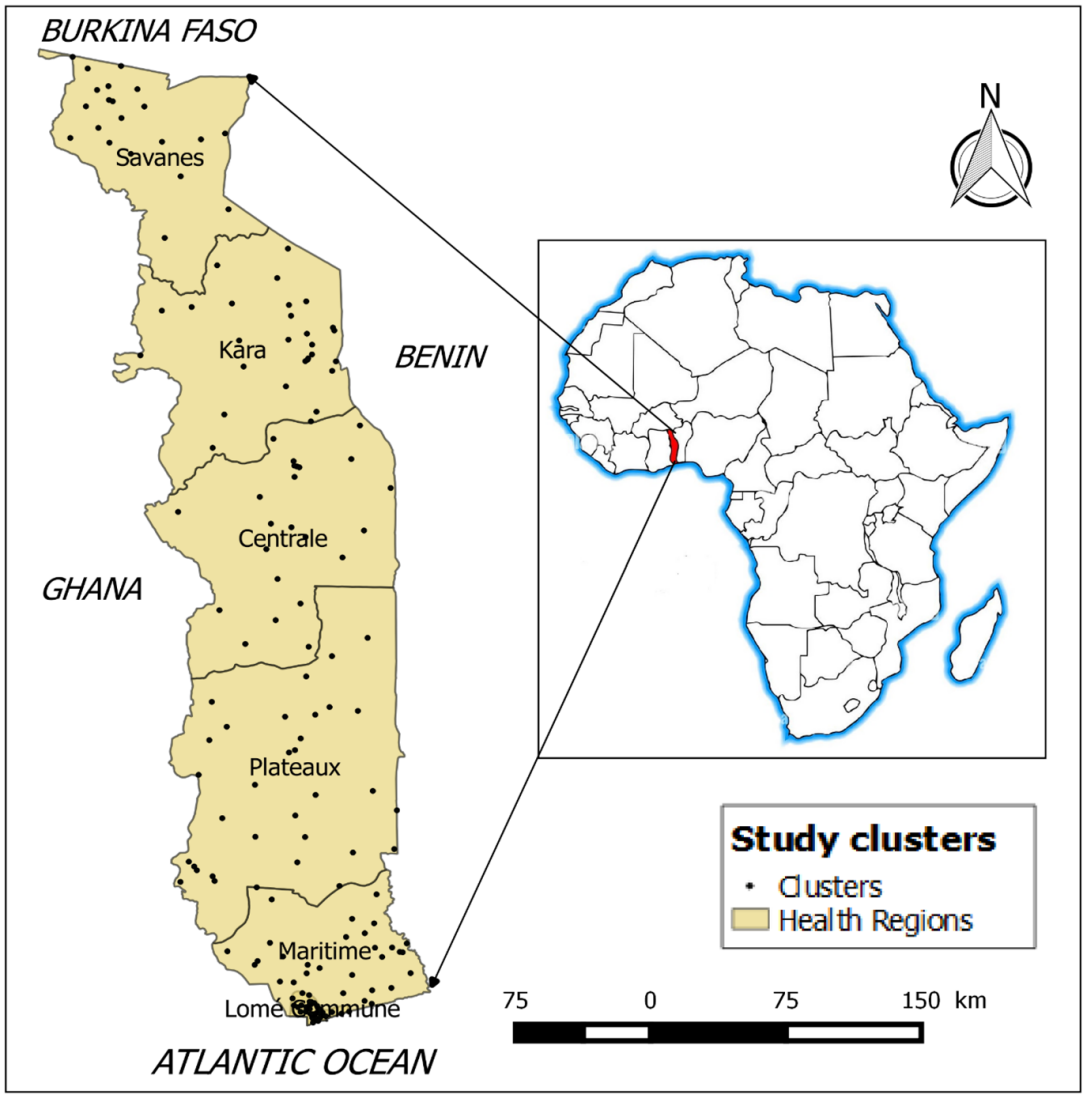
Study clusters (n = 171) of the 2017 Togo MIS. Source: Region level country shapefiles obtained from spatial reference; https://spatialreference.org/.

Togo is a country located in West Africa, on the coast of the Gulf of Guinea. It is bordered to the north by Burkina Faso, to the south by the Atlantic Ocean, to the east by Benin and to the west by Ghana (Fig. 1). In 2022, Togo had a population about 8 million inhabitants with a density of 152 inhabitants/km2. It covers an area of 56,785 km^2^ with 95.8% land and 4.2% water. It stretches over a length of 600 km and a width varying between 50 and 150 km. The highest altitude is at 986 m.

### Study design and data source

Cross-sectional data were collected as part of the Togo Malaria Indicator Survey (MIS) 2017–2018, which is a national representative survey. The main objective of this MIS was to obtain population-based estimates for malaria indicators such as prevalence and risk factors. Standardized household cluster sampling methods were applied. Figure 1 presents the map of the 171 study clusters where data were collected.

The distribution of study clusters reflected a higher concentration in more densely populated areas as sampling was proportional to population size. The data collected included geospatial covariates such as temperature, aridity, rainfall, precipitation, proximity to water and vegetation derived from various sources, at different levels of national coverage^25^.

Malaria incidence data were calculated as the mean number of people per year with fever, in the 2 km (urban) or 10 km (rural) buffer zone surrounding the survey cluster location. Cases obtained from each country surveillance system are reported by the National Malaria Control Program (NMCP). This includes among others information on the number of suspected cases, number of tested cases, Number of positive cases by method of detection and by species as well as number of health facilities that report those cases. This information is summarized in a District Health Information Software (DHIS2) application^25,26^. The DHS experts made a link between DHS data with routine health data, health facility locations, local infrastructure such as roads and rivers, and environmental conditions. Aside from this, data on malaria incidence was obtained from all 171 MIS clusters in the country with a 5 year interval (2000, 2005, 2010 and 2015). The Table 1 below presents the descriptions of the different variables included in the study.

**Table 1:** Overview of data sources for the climatic and environmental variables.

Variables	Description	Source
Temperature	Mean monthly temperature in °C	Global climate data; https://www.worldclim.org/
Aridity	Aridity index from driest to wettest	Global climate data; https://www.worldclim.org/
Rainfall	Average monthly rainfall in mm https://www.worldclim.org/	Climate hazards group infrared precipitation with stations (CHIRPS) https://www.chc.ucsb.edu/data/chirps
Precipitation	Precipitation seasonality	Global climate data; https://www.worldclim.org/
Proximity to water	Distance to water bodies in (km)	Food and agriculture organization http://www.fao.org/geonetwork/srv/en/main.home
Enhanced vegetation index (EVI)	Mean vegetation index value	US Geological Survey ; https://earlywarning.usgs.gov/
population density	Density related to count per square km	Gridded population of the world ; https://sedac.ciesin.columbia.edu/data/collection/gpw-v3
Altitude	Cluster altitude in meters	Gridded population of the world ; https://sedac.ciesin.columbia.edu/data/collection/gpw-v3

### Statistical analysis

Analyses of malaria incidence and climatic and environmental factors was performed through the computation of mean difference, frequency counts, percentages, independent t-test, and one-factor analysis of variance (ANOVA). Geospatial modelling exploring malaria incidence in relation to environmental and climatic variables, including temperature, rainfall, precipitation, distance to water bodies, aridity and population density were performed using the Ordinary Least Squares (OLS), the Spatial Lag Model (SLM), and the Spatial Error Model (SEM). The non-spatial OLS model provides a global model of the variable or process one is trying to understand or predict^27^; it describes the relationship between one or more quantitative variables and a dependent variable provides the covariate adjustment and prediction of mean risk of malaria in an area^28^. The spatial SLM model can accommodate a spatial dependency between the dependent variable and explanatory variables by incorporating a “spatially-lagged dependent variable” in the regression model. This model accounts for autocorrelation in the model with the weight matrix. The spatial SEM model is a model that considers corrective measures for residual spatial autocorrelation. For this model, the residuals were not assumed to be independent; instead, they exhibited a moving average map pattern^27,29^. The aim of using both spatial and non-spatial models is to compare the best-fitting models for predicting malaria incidence in children, using Akaike Information Criterion (AIC) to decide, and also to produce the most plausible risk maps^19,27,29^. The AIC allows us to select the model that minimizes the loss of information. We chose these models since we it to provide unbiased point estimates of the parameters. They offer the possibility to estimate spatial overlap coefficients, thus informing about correlation or spatial influence processes^30^.

Overall spatial autocorrelation between an ecological predictor and the incidence of malaria was assessed with Moran’s I statistical test^27^. The Moran scatter plot was used to quickly obtain an overview of the global spatial autocorrelation of malaria incidences, and a Local Indicators of Spatial Association (LISA) analysis was used to identify hot and cold spots of cluster location^27,28^. The analyses were performed using R software.

### Ethics approval and consent to participate

For this study, ethics approval was not sought since our analysis was based on publicly available data. However, DHS reports that informed consent, both written and verbal, was obtained from all participants by the Institutional Review Board of ICF International and the Bioethics Committee for Health Research (BCRS) of Togo. Prior to the start of the investigation, all ethical guidelines governing the use of human subjects were strictly adhered to and the methods were applied in accordance with the relevant guidelines and regulations of the Declaration of Helsinki. The data set and permission to conduct secondary data analysis were granted by the DHS program.

## Results

### Spatial and temporal distribution of the mean malaria incidence (per 1000 population at risk)

**Figure 2:**
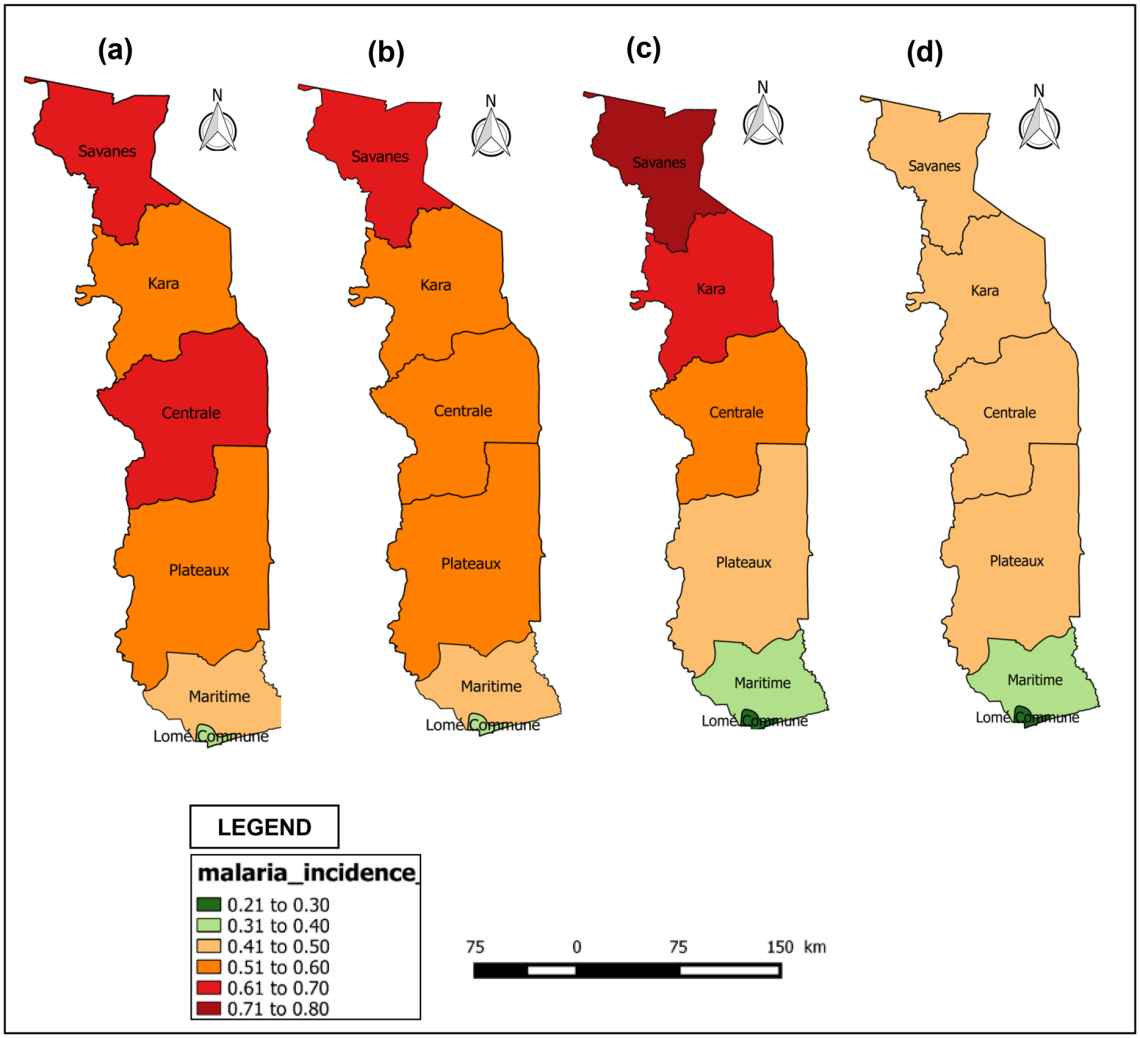
Spatio-temporal variation of malaria incidence in children under 5 years old in Togo: (**a**) Malaria incidence in 2000, (**b**) Malaria incidence in 2005, (**c**) Malaria incidence in 2010, (**d**) Malaria incidence in 2015. (Source: Region level country shapefiles obtained from spatial reference; https://spatialreference.org/)***.***

The spatial distribution of the mean malaria incidence in Togo in 2000, 2005, 2010 and 2015 is presented in Fig. 2 below. Overall, between 2000 and 2015, the mean malaria incidence decreased, although an increase in the average malaria incidence was observed in the Savanes region until 2010. The Savanes and Central health regions had a higher average malaria incidence than the Plateaux and Kara regions in 2000. The evolution of the incidence of malaria in 2010 shows an aggravation in the Savannah and Kara regions. The Lomé commune region recorded the lowest average malaria incidence rates in 2000, 2005, 2010 and 2015.

### Spatial autocorrelation analysis of malaria incidence

The effects of the most important environmental and climate factors as described in the methods are presented in Table 2. Both climatic and environmental factors, in particular temperature, aridity, precipitation and proximity to water, were related to the mean malaria incidence in each of the three analysis models.

**Table 2:** Spatial models showing correlation between the mean malaria incidence and climatic and environmental variables.

Predictor	Ordinary least square (OLS)		Spatial lag model (SLM)		Spatial error model (SEM)	
	Coefficient (%)	*P-value*	Coefficient (%)	*P*-value	Coefficient (%)	*P*-value
Intercept	− 5.433	0.021	− 5.433	0.000	− 5.589	0.000
Mean temperature	0.552	0.032	0.544	0.001	0.564	0.000
Aridity	0.650	0.014	0.693	0.000	0.706	0.000
Rainfall	− 0.024	0.366	− 0.026	0.134	− 0.026	0.145
Annual precipitation	0.392	0.002	0.414	0.000	0.423	0.000
Proximity to water	0.007	0.062	0.010	0.001	0.006	0.012
Enhanced vegetation index	0.010	0.690	0.011	0.868	− 0.004	0.948
Population density	− 0.026	0.511	− 0.019	0.041	− 0.027	0.341
Cluster altitude	0.013	0.981	0.015	0.882	− 0.013	0.975
AIC	− **51.436**		− **65.906**		− **48.437**	
Adjusted R^2^	0.675		0.827		0.813	

Significant values are in bold.

The SLM model with the lowest AIC (− 65.906) was the best model with the best fit. For mean temperature, there was a positive significant association with malaria incidence in children under five. Each additional one-degree Celsius increase in mean temperature (26–29 °C) being associated with an increase in incidence of about 0.55%. For precipitation, with every millimeter of additional water precipitated, there was an associated increase in malaria incidence of the order of 0.392% in Togo. Malaria incidence also increased with an increase in aridity index and with proximity to water. In contrast, annual rainfall and population density might bean inversely associated with mean malaria incidence.

### Spatial autocorrelation: global moran’s I

**Figure 3:**
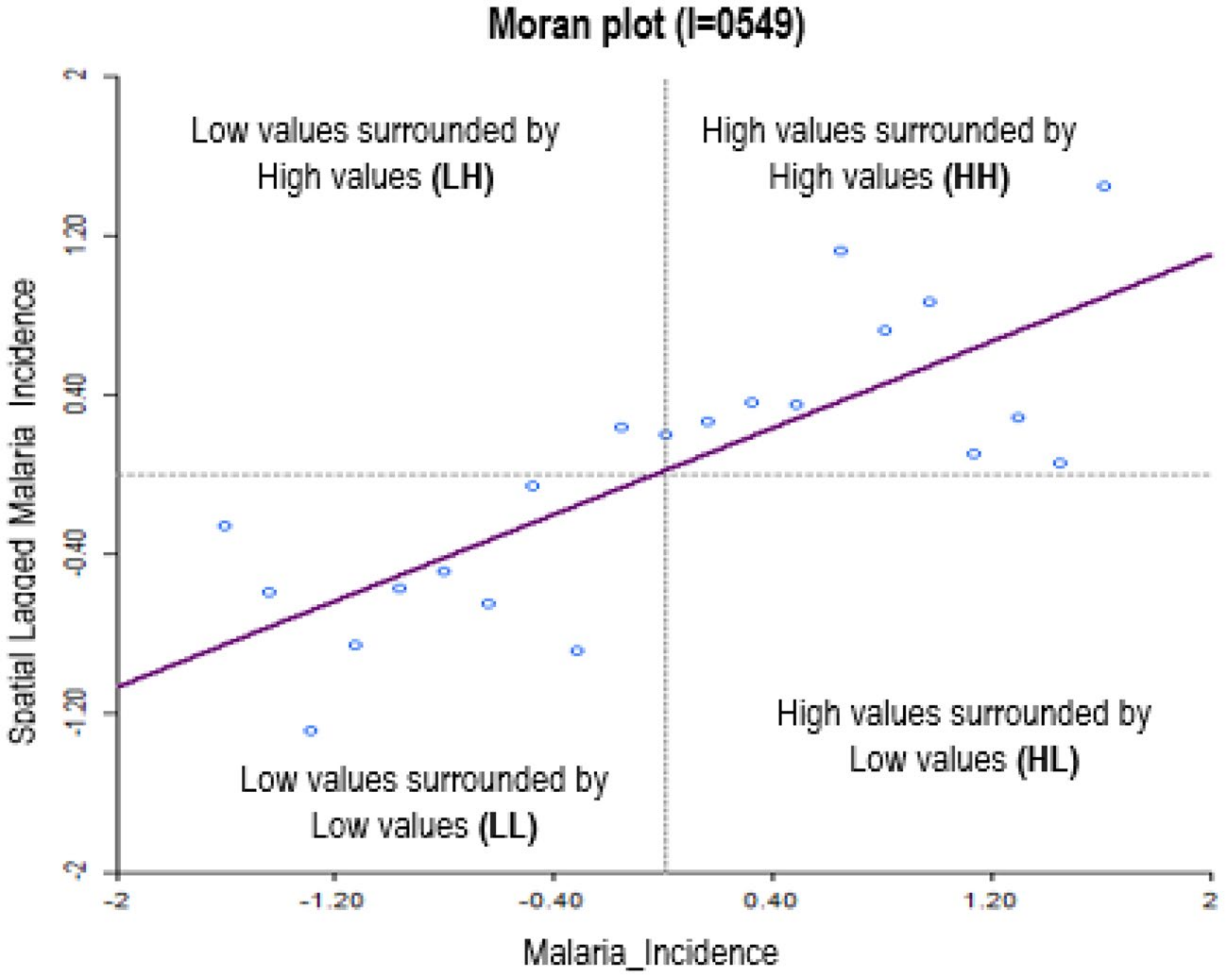
Mean Moran’s I values for local spatial autocorrelation for malaria incidence at varying spatial lags.

The global Moran's I test (Moran I = 0.469, p < 0.05) showed significant spatial dependence in the observed malaria incidences. Figure 3 depicts a scatter plot of mean malaria incidence.

### Spatial autocorrelation: local indicators of spatial association (lisa)

**Figure 4:**
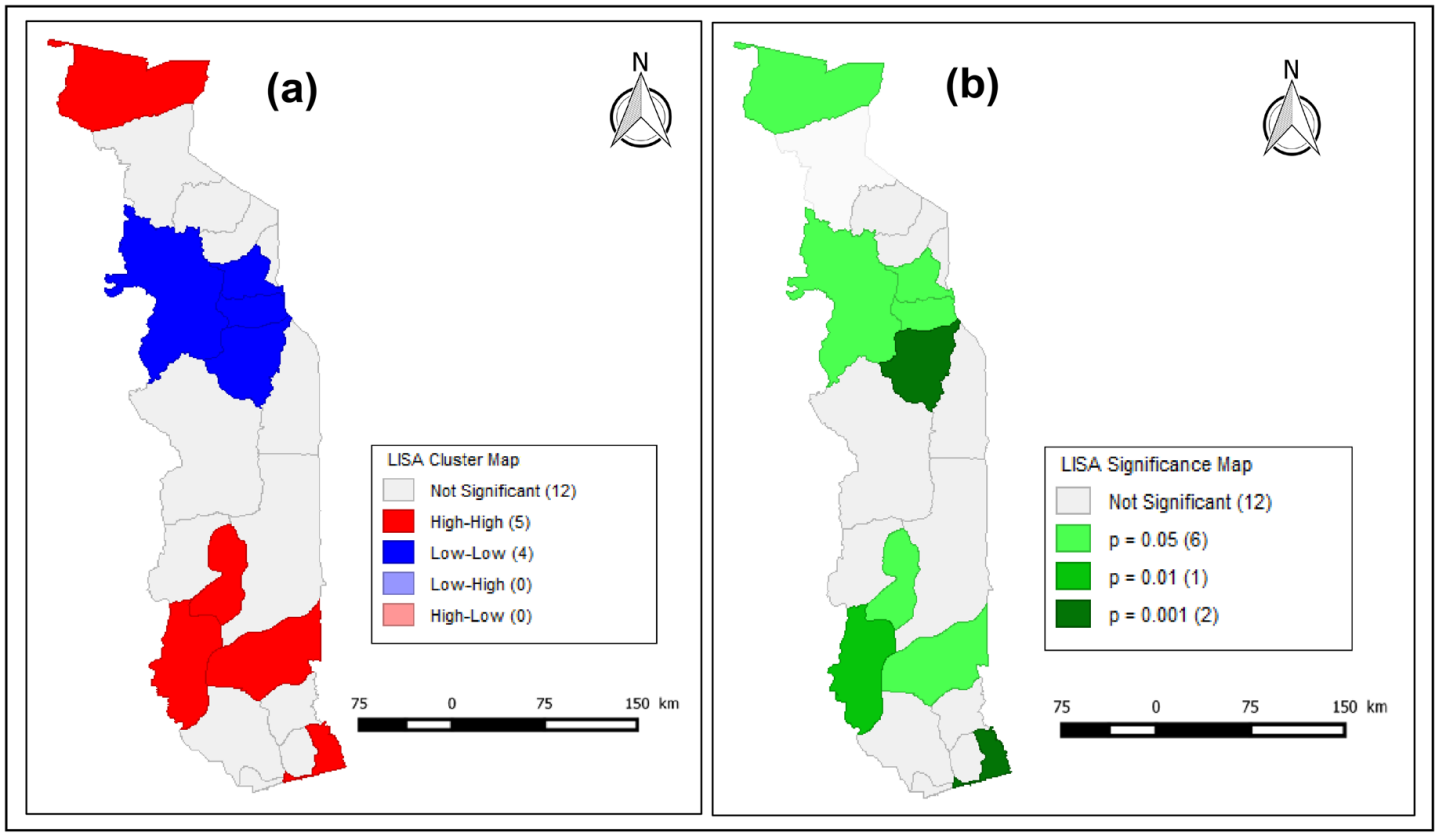
(**a**) LISA map (**b**) and LISA significance map of Local Moran’s I test show the hot and cold malaria spot locations in Togo using GeoDa software.

The LISA results are shown in Fig. 4 below.

The areas shown in red in Fig. 4a are the locations of clusters representing hot spots. The areas shown in blue are the locations of clusters representing cold spots. Figure 4b shows the significance levels, with different colors representing different p-value ranges. Dark green represents areas with significant (p-value less than 0.001) local spatial autocorrelation in malaria incidence; simple green represents areas with significant (p-value less than 0.01) local spatial autocorrelation in malaria incidence; and bright green represents areas with significant (p-value < 0.05) local spatial autocorrelation in malaria incidence.

## Discussion

Malaria remains a serious threat in sub-Saharan Africa, including Togo, even if incidence has decreased overall, with significant differences in incidence between regions over the years. This study used spatial and non-spatial regression models to identify hotspots of malaria infection transmission in Togo through ecological analysis. Spatial heterogeneity could be explained by the precipitation seasonality, mean temperature, aridity index and proximity to water which were all positively correlated with malaria incidence in our analyses. All these factors are predominant environmental factors that characterized the Plateaux region where the highest incidence was observed and favour malaria transmission in the region^10^.

For all the ecological predictors, some, as mentioned above (the mean temperature, aridity, precipitation seasonality and proximity to water), showed positive correlations and significant associations with malaria incidence. The daily survival rate of mosquitoes is optimal at about 90% with temperatures between 16°C and 36°C (Craig 1999). Increasing temperature reduces the blood meal-seeking behaviour of female Anopheles mosquitoes, resulting in a corresponding decrease in ovulation and juvenile mosquito production, and consequently a decrease in the number of new malaria cases. Increased precipitation was also positively associated with malaria incidence and can have both a direct and indirect effect, particularly where dams are located. Precipitation increases reservoir water levels and creates potential breeding grounds for mosquitoes along the banks of the reservoir^12,31^. The southern regions (Lomé commune, Maritime, Plateaux and Central) benefit from a longer duration of rainfall than the northern regions (Kara and Savanes) in Togo^10^. Concerning aridity, this very strong positive correlation with malaria incidence may be related to the fact that increased aridity influences malaria transmission by reducing the mosquito biting rate and adult life span as well as the extrinsic incubation period of the malaria parasite^18,31^. Thus, the ability of adult vectors to survive long enough and contribute to the spread of parasites, and the ability of pre-adult stages to sustain a minimum population, depends on aridity levels and species-specific resilience to arid conditions^32^. In contrast, a study in northeastern Benin^33^ georeferencing all mosquito breeding sites in two rural sites showed that aridity had a negative influence on malaria transmission. According to the authors, this could be due to the fact that classic anopheles breeding sites have dried up due to the very high aridity in these regions, leading gravid females to lay their eggs in unusual habitats already hosting larvae of other general species. As for proximity to water bodies, our results corroborate with studies in Nigeria^19^ and The Gambia^34^. Using respectively logistic regression methods and post-hoc analyses on mosquitoes catches, these studies observed a positive relationship with distance to water bodies up to 4 km before decreasing.

Other ecological predictors showed inverse but non-significant relationships, such as rainfall and population density. The inverse correlation with rainfall observed in the three analysis models suggests a negative impact of continuous heavy rainfall on malaria parasite vectors and eventual transmission of the disease. Similar previous studies conducted in Burkina Faso^10,31,32^ also showed a negative correlation of rainfall on malaria incidence. Another study in Sri Lanka^35^ observed seasonal variation in malaria infection in the country. The varying seasonal effects of rainfall on the number of malaria cases was reflected in the weak correlations observed, in which situation rainfall may be of limited use in predicting malaria. An observed linear relationship between rainfall and malaria could hide non-linear effects. An important aspect would be to investigate the relationship between rainfall and mosquito reproduction and survival. Variables influenced by rainfall, such as soil water saturation and river flow, could be more directly linked to the specific breeding conditions of malaria vectors. However, these variables are more costly to measure and are therefore often estimated from rainfall^17^.

Prevention measures are also likely to have a significant temporal effect on the time series of malaria cases. E.g. a study in urban and peri-urban areas in Africa (Mozambique and Senegal) and in the Indian Ocean (republic of Mauritius)^36^ found a negative association between malaria infection and vector abundance. They attributed this to the willingness of people to use mosquito nets or to the stimulated development of immunity during early childhood in high-risk areas^37^. This variable has not been considered. Moreover, control methods and insecticides have changed over time, making it a complex variable^35^.

A limitation of spatial statistical models is that disease data can show a great deal of intra-and inter-annual variability, while a regression analysis assumes that the association between exposure and outcome is stationary over time^29^. Generalized linear mixed models (GLMM) could be used to capture transmission dynamics over time while controlling for temporal autocorrelations^38^.

Nevertheless, the data illustrate the opportunity to identify hotspots for malaria control. With regard to seasonal climate forecasts, environmental monitoring and the evolution of malaria morbidity in the country, these results can help design an early warning system for the National Malaria Control Program, which will help identify areas at risk of epidemic outbreak.

## Conclusion

We evaluated the spatial and temporal association in Togo between malaria transmission hotspots and environmental and climatic risk factors. Malaria incidence was more related to incidence in nearby clusters than to those far away. Mean temperature, precipitation, aridity and proximity to water bodies showed a significant and positive association with malaria incidence. These results may help to channel available resources to disease hotspots and sustain prevention efforts for better control of malaria infection in children under 5 years of age in Togo and in similar sub-Saharan context.

## Data Availability

DHS datasets are publicly available on www.dhsprogram.org.

## References

[1] World Health Organization. World malaria report 2022. [cité 11 juin 2023]. World malaria report 2022. Disponible sur: https://www.who.int/teams/global-malaria-programme/reports/world-malaria-report-2022. (2022).

[2] Ministry of Health and Public Hygiene. Ministère de la Santé et de l’Hygiène Publique. [cité 2 août 2022]. Plan Stratégique National de Lutte contre le Paludisme 2021–2025. Disponible sur: https://www.sante.gov.bf/ressources/documents. (2022).

[3] Kombate G, Cakpo GE, Azianu KA, Labité MA, van der Sande MAB (2022). Care-seeking behaviour among febrile children under five in Togo. BMC Public Health.

[4] Kombate G, Gmakouba W, Scott S, Azianu KA, Ekouevi DK, van der Sande MAB (2022). Regional heterogeneity of malaria prevalence and associated risk factors among children under five in Togo: evidence from a national malaria indicators survey. Malar J..

[5] Bakai TA, Thomas A, Iwaz J, Atcha-Oubou T, Tchadjobo T, Khanafer N (2020). Changes in registered malaria cases and deaths in Togo from 2008 to 2017. Int. J. Infect. Dis..

[6] Dorkenoo AM, Gbeasor-Komlanvi FA, Gbada K, Zida-Compaore WIC, Teou D, Konu YR (2022). Prevalence of malaria and covid-19 in febrile patients in Lomé, Togo in 2020. Acta Parasitol. sept.

[7] Koudaya YE, Ahadji-Dabla KM, Koffi E, Tatah PP, Ketoh GK (2022). Indicators and vectors related to malaria transmission in the Kozah and Doufel gou (Kara region, North Togo). Int. J. Biol. Chem. Sci..

[8] Djadou KE, Takassi EO, Guédénon JK, Atakouma YD. Severe malaria in children at Tsevie hospital (Togo). In: *2017 International Rural and Elderly Health Informatics Conference (IREHI).* 1‑4. (2017).

[9] Landoh ED, Tchamdja P, Saka B, Tint KS, Gitta SN, Wasswa P (2012). Morbidity and mortality due to malaria in Est Mono district, Togo, from 2005 to 2010: a times series analysis. Malar J..

[10] Djame Y, Lare LY, Djangbedja M (2018). Variabilité climatique et épidémiologie du paludisme dans la région des Savanes au Nord-Togo. J Rech Sci L’Université Lomé.

[11] Ministère de la santé et de la protection sociale. Plan Stratégique National (PSN) et du Plan de suivi-évaluation (PSE) 2017–2022 [Internet]. Lome, Togo: Ministère de la santé et de la protection sociale; [cité 11 juin 2023] p. 93. Disponible sur: https://sante.gouv.tg/wp-content/uploads/2021/06/Plan-Suivi-Evaluation_PNDS-2017-valide-13-09-17.pdf. (2017).

[12] Okunlola OA, Oyeyemi OT (2019). Spatio-temporal analysis of association between incidence of malaria and environmental predictors of malaria transmission in Nigeria. Sci. Rep..

[13] Patz JA, Olson SH (2006). Malaria risk and temperature: Influences from global climate change and local land use practices. Proc. Natl. Acad. Sci..

[14] Ilboudo-Sanogo E, Tiono BA, Sagnon N, Cuzin Ouattara N, Nébié I, Sirima SB (2010). Temporal dynamics of malaria transmission in two rural areas of Burkina Faso with two ecological differences. J. Med. Entomol..

[15] Carter R, Mendis KN (2002). Evolutionary and historical aspects of the burden of Malaria. Clin. Microbiol. Rev..

[16] Debebe Y, Hill SR, Tekie H, Dugassa S, Hopkins RJ, Ignell R (2020). Malaria hotspots explained from the perspective of ecological theory underlying insect foraging. Sci. Rep..

[17] Haque U, Hashizume M, Glass GE, Dewan AM, Overgaard HJ, Yamamoto T (2010). The role of climate variability in the spread of malaria in Bangladeshi highlands. PloS One..

[18] Shililu J, Ghebremeskel T, Mengistu S, Fekadu H, Zerom M, Mbogo C (2003). High seasonal variation in entomologic inoculation rates in Eritrea, a semi-arid region of unstable malaria in Africa. Am. J. Trop. Med. Hyg..

[19] Onyiri N (2015). Estimating malaria burden in Nigeria: A geostatistical modelling approach. Geospat. Health.

[20] McMahon A, Mihretie A, Ahmed AA, Lake M, Awoke W, Wimberly MC (2021). Remote sensing of environmental risk factors for malaria in different geographic contexts. Int. J. Health Geogr..

[21] Rouamba T, Nakanabo-Diallo S, Derra K, Rouamba E, Kazienga A, Inoue Y (2019). Socioeconomic and environmental factors associated with malaria hotspots in the Nanoro demographic surveillance area Burkina Faso. BMC Public Health.

[22] Malaria hotspots and climate change trends in the hyper-endemic malaria settings of Mizoram along the India–Bangladesh borders | Scientific Reports [Internet]. [cité 2 janv 2024]. Disponible sur: https://www.nature.com/articles/s41598-023-31632-6?error=cookies_not_supported&code=b808cb49-d92f-4f74-8326-4c63f2bf815f. (2024). 10.1038/s41598-023-31632-6 PMC10025798 36941291

[23] Odhiambo JN, Kalinda C, Macharia PM, Snow RW, Sartorius B (2020). Spatial and spatio-temporal methods for mapping malaria risk: a systematic review. BMJ Glob Health.

[24] Zhou G, Minakawa N, Githeko A, Yan G (2004). Spatial distribution patterns of malaria vectors and sample size determination in spatially heterogeneous environments: A case study in the West Kenyan Highland. J. Med. Entomol..

[25] DHS Program. Geospatial Covariate. [cité 11 juin 2023]. Spatial Data Repository - Geospatial Covariates. Disponible sur: https://spatialdata.dhsprogram.com/covariates/. (2023).

[26] Mayala BK, Donohue RE, Dontamsetti T, Fish TD, Croft TN (2022). Interpolation of DHS survey data at subnational administrative level 2. Stat. J. IAOS.

[27] Beale CM, Lennon JJ, Yearsley JM, Brewer MJ, Elston DA (2010). Regression analysis of spatial data. Ecol. Lett..

[28] Kleinschmidt I, Bagayoko M, Clarke GP, Craig M, Le Sueur D (2000). A spatial statistical approach to malaria mapping. Int. J. Epidemiol..

[29] Arifin SMN, Davis GJ, Zhou Y. Modeling space in an agent-based model of malaria: comparison between non-spatial and spatial models. In Proc. of the 2011 Workshop on Agent-Directed Simulation. 92-9 (San Diego, CA, USA: Society for Computer Simulation International, 2011).

[30] Rüttenauer T (2022). Spatial regression models: A systematic comparison of different model specifications using monte carlo experiments. Sociol. Methods Res..

[31] Kibret S, Glenn Wilson G, Ryder D, Tekie H, Petros B (2019). Environmental and meteorological factors linked to malaria transmission around large dams at three ecological settings in Ethiopia. Malar J..

[32] Guerra CA, Gikandi PW, Tatem AJ, Noor AM, Smith DL, Hay SI (2008). The limits and intensity of plasmodium falciparum transmission: Implications for malaria control and elimination worldwide. PLoS Med..

[33] Govoetchan R, Gnanguenon V, Ogouwalé E, Oké-Agbo F, Azondékon R, Sovi A (2014). Dry season refugia for anopheline larvae and mapping of the seasonal distribution in mosquito larval habitats in Kandi, northeastern Benin. Parasit Vectors.

[34] Thomas CJ, Cross DE, Bøgh C (2013). Landscape movements of Anopheles gambiae malaria vector mosquitoes in rural Gambia. PloS ONE..

[35] Briët OJ, Vounatsou P, Gunawardena DM, Galappaththy GN, Amerasinghe PH (2008). Models for short term malaria prediction in Sri Lanka. Malar J..

[36] Carter R, Mendis KN, Roberts D (2000). Spatial targeting of interventions against malaria. Bull. World Health Organ..

[37] Thomson MC, Connor SJ, Milligan P, Flasse SP (1997). Mapping malaria risk in Africa: What can satellite data contribute?. Parasitol. Today.

[38] Kleinschmidt I, Sharp BL, Clarke GPY, Curtis B, Fraser C (2001). Use of generalized linear mixed models in the spatial analysis of small-area malaria incidence rates in KwaZulu Natal. South Afr. Am. J. Epidemiol..

